# Influence of fiber dimensions on the mechanical properties of silica glass nanofibers

**DOI:** 10.1186/s11671-025-04210-0

**Published:** 2025-02-14

**Authors:** Raúl Barciela, Félix Quintero, Thiruvilla S. Mahadevan, Antonio Riveiro, Juan Pou, Jincheng Du

**Affiliations:** 1 LaserON Research Group, CINTECX, E.E.I, Universidade de Vigo, 36310 Vigo, Spain; 2 Department of Materials Science and Engineering, University of North Texas, Denton, TX 76203 USA; 3 Materials Engineering, Applied Mechanics and Construction Department, E.E.I., University of Vigo, 36310 Vigo, Spain

## Abstract

The understanding of the mechanical properties in glass nanofibers remains a challenge. As the fiber diameter decreases, surface-to-volume atom fraction increases, making the impact of surface defects more significant. To elucidate these effects, we employed classical molecular dynamics (MD) simulations to investigate how fiber dimensions and the surface layer influence the mechanical properties of silica glass nanofibers. Our simulation methodology included fibers of varying diameters, generated using two different production methods (i.e. “cutting” and “casting” methods) that produce different degrees of surface atomic defects, and compared with bulk samples without surface atoms. The defect-rich surface layer of these fibers was carefully analyzed. Then, MD tensile simulations were performed to analyze the effect of the fiber surface on the mechanical properties and to explain the onset of the brittle-to-ductile transition experimentally observed at a few tens of nanometers. The results revealed that the surface layer maintains a fixed thickness independent of the fiber diameter, resulting in a pronounced increase of the fiber defects in thin fibers. Also, the tensile test simulations show that surface defects significantly reduce tensile strength, without appreciably increasing ductility compared to bulk samples. In turn, we show that the brittle-to-ductile transition is not caused by the surface defects, but related to a balance between fracture energy and elastic energy, which varies with fiber length. Using experimental values of different glass properties, our theory predicted a threshold length of around 200 nm, below which ductile fracture dominates, in reasonable agreement with experimental results.

**Graphic abstract:**

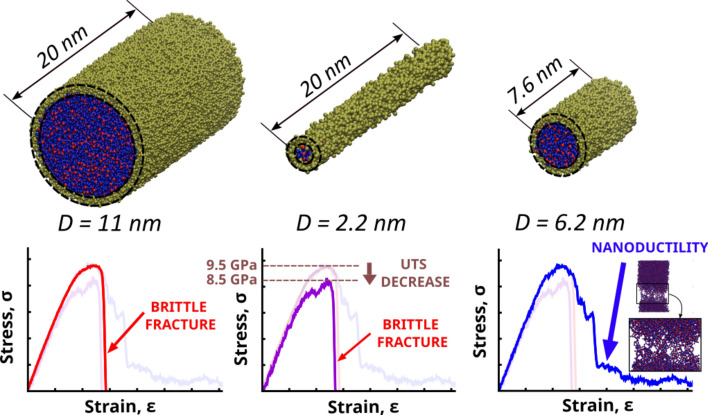

**Supplementary Information:**

The online version contains supplementary material available at 10.1186/s11671-025-04210-0.

## Introduction

Advancing the mechanical performance of glasses involves the quest to combine high strength and enhanced toughness. However, traditionally, a compromise between these two properties has been a challenge, as high strength materials typically show brittle fracture hence low toughness due to low or absence of plasticity (e.g. ceramics). Other materials show an extended ductility and high toughness but relatively moderate strength (e.g. metals).

In some amorphous materials such as bulk metallic glass, a competitive combination of strength and ductility can be achieved at the micrometric and nanometric ranges, taking advantage of size effects [1–7]. Studies on tensile deformation of metallic glasses have shown that, just by reducing dimensions below 100 nm, the material can achieve large, metal-like, ductility (above 25%) [2, 8, 9], while retaining a ceramic-like strength (above 2 GPa) [2, 9]. In silica glass nanofibers, outstanding strength values above 10 GPa have been reported [6, 7], approaching that obtained in glass fibers tested under controlled environments [10, 11]. Also, several works evidenced a pronounced ductile deformation in silica glass nanofibers under tensile stress [7, 12, 13]. While some of these results on silica glass nanofibers have been attributed to the electron beam effects during in situ tensile tests in TEM [12], beam-off experiments confirm that ductility can also be induced without the electron beam influence [7, 13]. Indeed, Luo et al. reported fracture strains up to 18% in amorphous silica nanowires of diameters less than 10 nm under beam-off conditions [7]. These promising results make glass nanofibers very attractive towards multiple applications, such as reinforcing agents in high mechanical performance nanocomposites or components of photonic circuits and nanoelectronic devices.

Intensive experimental [5–7, 12–14] and simulation [15–18] work has been performed and several interpretations have been proposed to understand the fracture mechanisms and mechanical properties of silica and silicate glasses at the nanoscale. A preliminary interpretation for the increase of strength in the micro- and nanoscale has traditionally invoked the extreme value theory (EVT) [19] and Griffith fracture mechanics [20]. Accordingly, size effects lead to the unavoidable constraint in the size and frequency of the flaws, which reduces the probability of stress concentrations and provides another means of approaching intrinsic strengths at the nanoscale [6, 21].

However, the understanding of the ductile processes in glass nanofibers has been a longstanding challenge [1, 7, 17, 18]. Indeed, despite theories of failure of brittle solids as EVT may explain the increase of glass strength, owning to their simplicity, they are not convincing to explain the fracture behavior of ductile glasses [1], and, more importantly, the brittle-to-ductile transition (BDT) observed in glass nanofibers at the nanoscale [7]. In this regard, atomistic simulations have become valuable tools to describe structure–property relations in many materials [22, 23], and, particularly in glasses and glass fibers [24, 25]. They have the capability to generate amorphous structures and perform mechanical testing with different boundary conditions, which allow for isolating the fiber surface and analyzing its role on the mechanical properties [17].

Two opposed theories about nanoscale ductility can be distinguished in the literature. First theory, supported by atomistic simulations, suggested that nanoscale ductility may be associated with surface effects [7, 26–28]. Indeed, in glass nanofibers, ductility was associated with a defect-rich surface layer, where the abundant undercoordinated atoms display a much higher mobility than in the bulk [7, 29]. This mobility allows to mediate fast surface repair mechanisms, resembling thermally activated viscous flow. Therefore, the defect-rich surface layer that enables nanoscale plastic deformation has been commonly proposed as the triggering mechanism for the BDT observed in silica glass nanofibers [7].

The second and more recent theory challenges the surface origin of nanofiber ductility, suggesting that ductile processes can occur in bulk amorphous structures in the absence of surfaces. [5, 15, 16, 30]. Instead, experiments have shown that nanoductility may appear in localized regions due to the heterogeneous character of the atomic network and rigidity fluctuations at the nanoscale [15, 16]. Atomistic simulations also revealed that ductile behavior can exist in bulk glasses at sufficiently small scales [31, 32]. It was also suggested that ductility may arise from cumulative damages in the bulk during deformation, which becomes relevant at the nanoscale, rather than surface effects [17].

Therefore, the factors involved in the fracture behavior and mechanical properties of glass nanofibers still remain unclear. Specifically, whether the ductile behavior of silica glass fibers is caused by damage accumulation in the bulk amorphous structure or the surface plastic effect is still controversial, leaving the explanation of the intriguing BDT transition in silica nanofibers unresolved [7]. As a result, a more comprehensive study is needed to disentangle the roles of structure size and surface plasticity in the BDT observed in experimental works.

To solve this puzzle, we performed classical molecular dynamics (MD) simulations to provide an atomistic description of the deformation of silica nanofibers with the aim to isolate the role of surface defects by generating structures with different boundary conditions. Using two different nanofiber production methods that generate fiber models with different degrees of surface defects, we perform extensive MD simulations of tensile deformation to study the role of the surface layer defects and fiber dimension on the atomic scale fracture behaviors. Particularly, we characterize the surface layer in terms of atomic defects and thickness. Then, we perform MD tensile tests to analyze the effect of the fiber surface on the mechanical properties and deformation behavior. Finally, we attempt to explain the onset of the experimentally observed BDT in silica glass nanofibers. Contrary to previous findings, we demonstrate that the surface defects effect is not the driver of the BDT. Conversely, we find a reasonable correlation of the onset of BDT with fiber dimensions and aspect ratios. To shed light on this effect, we propose a phenomenological model for size dependence based on the competition between two energy terms (i.e. elastic energies and fracture energies).

## Simulation methods

### Atomic interactions

Silica glass fiber simulations were performed using the large-scale atomic/molecular massively parallel simulator (LAMMPS) package [33]. Atomic interactions were described using three different interaction potentials. The diffusive charge reactive potential (DCRP), developed by Mahadevan and Garofalini to study glass-water interactions in silica interfaces, is used as the reference potential in this work [34, 35]. This potential has the capability to model the reaction with water hence the effect of environment on the fiber mechanical properties. Our earlier work showed its capability to generate silica glass structures in good agreement with experiments such as neutron diffraction and model glass deformation behaviors [32]. Another potential employed here is a partial charge pairwise potential initially parametrized by D. M Teter and later improved by Du and Cormack, known as Teter potential [36]. The last potential considered is the widely used three-body Vashishta potential for pure amorphous silica [37]. In this work, the DCRP potential was selected to perform all simulations presented in the main text. The Teter and Vashishta potential were also employed to compare the results of some simulations which are presented in the Supporting Information.

### Modelling and simulation details

**Fig. 1:**
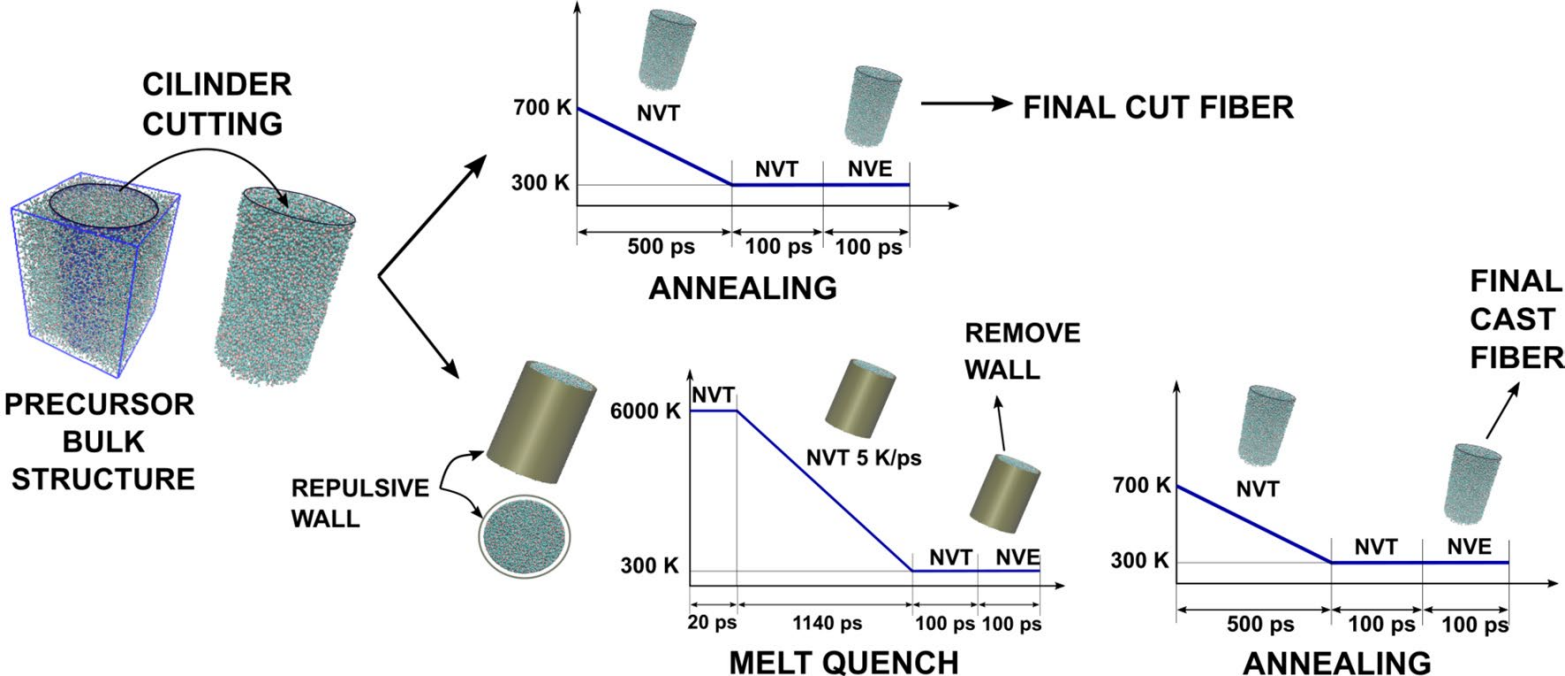
Process of generation of amorphous silica nanofibers following “cutting” and “casting” production methods

The fibers were prepared by using two simulation methods: the “cutting” method that generates fibers from bulk silica glass and the “casting” method that performs melt-and-quench in a cylindrical mold [31, 38, 39]. Figure 1 shows the simulation process for the generation of both fiber models. A precursor bulk amorphous silica structure was obtained through a simulated melt-and-quench process at a cooling rate of 5 K/ps (refer to Supporting Information for preparation details). The structural analysis of the generated structures, including their structure factors and pair distribution functions, shows that the structures generated using the three potentials are amorphous and in good agreement with experimental neutron diffraction data and results from solid state NMR spectroscopy (see Fig S1 for the structure factor and pair distribution function discussion).

Following the proposed approach, cylindrical glass shapes were cut from the precursor bulk amorphous silica structures stabilized at room temperature. After that, for the “cutting” method, the cut cylindrical structures where directly annealed and relaxed from 700 to 300 K in an NVT ensemble during 500 ps. Finally, after two equilibration steps of 100 ps at a constant volume (NVT ensemble) and constant energy (NVE ensemble), the cut fibers are obtained.

For the “casting” method, the cylindrical shapes were quenched from a melt while confined inside a repulsive cylindrical wall. A harmonic force field of the type ε(r-r_c_)^2^ was chosen to confine the atoms inside the wall. The parameters ε and the cut-off distance r_c_ where set to 1000 kcal/mol and 1 Å, respectively. The radius of the wall was chosen to be 2 Å higher than that of the cut cylindrical structures. Then, the structures were melted inside the wall to 6000 K for 20 ps in an NVT ensemble. After that, they were cooled continuously to 300 K in an NVT ensemble under a cooling rate of 5 K/ps. At room temperature, two subsequent equilibration steps of 100 ps at a constant volume and constant energy were performed. After that, the cylindrical wall was removed and the structures were annealed at 700 K, following the same procedure as for the cut fibers. After the annealing process, the cast fibers for subsequent deformation are obtained (Fig. 1). A time step of 1 fs was used for the integration of the trajectories, while the thermodynamic properties where extracted each 1000 fs.

In order to analyze the effect of the fiber production method on the deformation behavior, samples with varying diameters, *D*, ranging from 2.2 to 11.1 nm and a fixed length, *L*, of 20.1 nm, were generated following both production methods (Table 1). For each size, a different number of replicates, N_runs_, were generated using different initial random structures. In the larger samples, due to the dramatic increase of the computation time, N_runs_, is reduced. This choice does not compromise the statistical significance of the results. Indeed, in larger samples, the stochastic nature of atomic movement is less pronounced, yielding more consistent deformation behaviors across simulations. Therefore, fewer replicates are needed to achieve statistical significance due to the reduced variability in the larger sample tests.

**Table 1:** Dimensions (length, L, and diameter, D); number of atoms, N_at_, of the simulated fiber samples, and N_runs_, number of replicates for each size

Sample	*L*/nm	*D*/nm	*N*_at_	*N*_runs_
1	20.1	2.2	5763	10
2	20.1	3.3	12,972	10
3	20.1	5.0	29,112	10
4	20.1	8.4	81,045	5
5	20.1	11.1	144,096	3

### Pbc structures preparation

For a better comprehension of the role of surface in the fiber deformation, structures lacking surface atoms were also generated by imposing periodic boundary conditions (pbc) along all directions (pbc structures). The pbc structures were obtained starting from an orthogonal box with dimensions *M* × *M* × *L*. The structures have the same length *L* along the deformation axis and cross-sectional area *M*^2^ as the fiber models. Different equivalent pbc samples were generated for each of the different produced fibers. The protocol used to generate the pbc structures follows the same process as the one used to generate the precursor bulk amorphous silica structures.

### Uniaxial deformation tests

Uniaxial tensile deformations of the fibers and the pbc structures were performed at 300 K under a constant “engineering” strain rate of 10^9^ s^−1^ along the *z* direction. For the pbc structures, the atoms bounding the lateral faces were allowed to relax in NPT ensemble at a fixed pressure of 1 bar. Nosé-Hoover thermostats and barostats were used to control pressure and temperature during deformation [40–42]. First, the actual force acting on the fiber was calculated. Then, an engineering stress was obtained by dividing the instant force by the initial fiber cross section.

## Results and discussion

### The roles of fiber diameter and surface defects

**Fig. 2:**
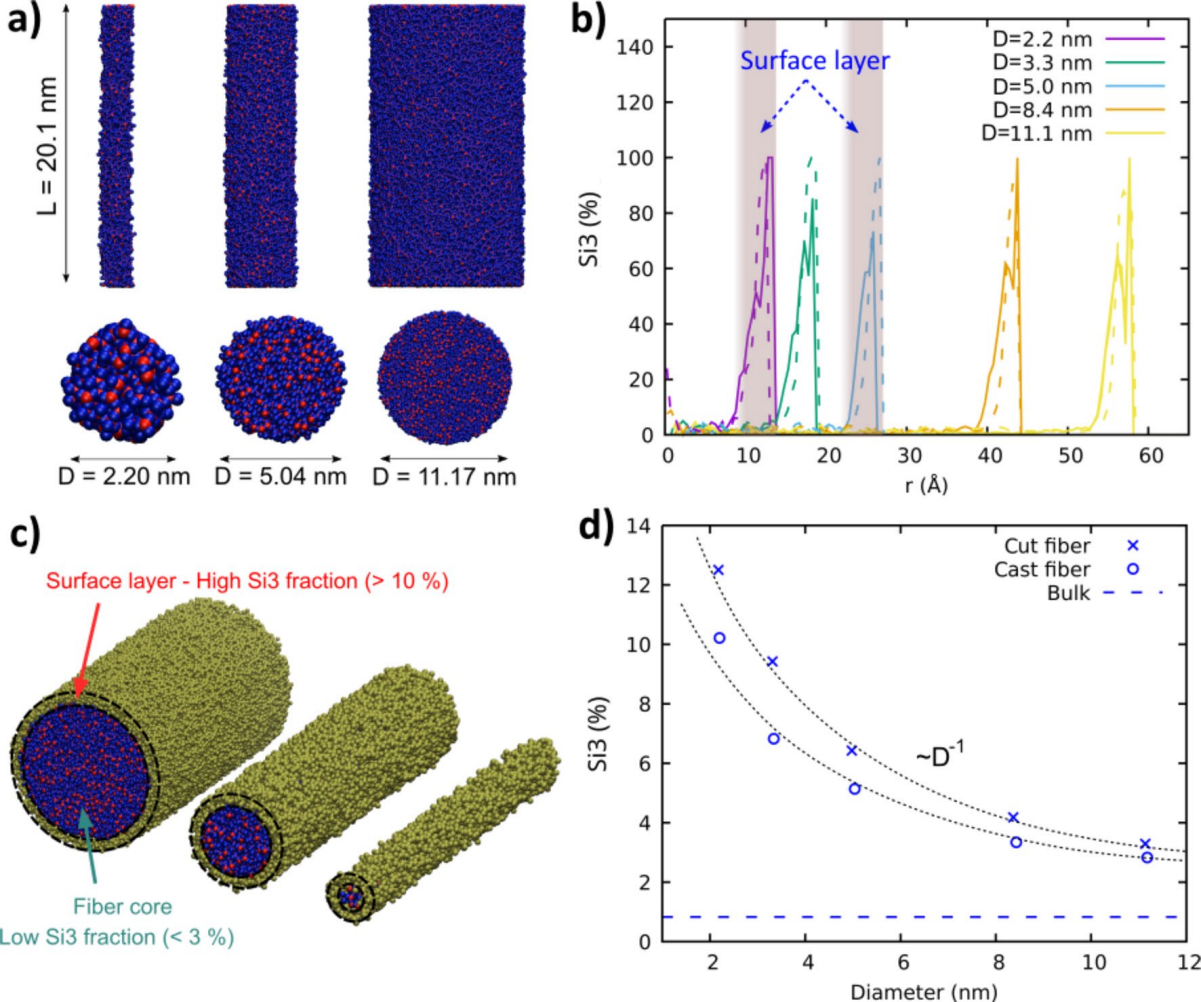
**a** Longitudinal views (upper panels) of amorphous silica fibers with different diameters generated using cast method and their respective cross-sectional views (bottom panels). Red and blue spheres represent silicon and oxygen atoms, respectively. **b** Radial distribution of Si3 fraction for the “cutting” (dashed lines) and “casting” (solid lines) production methods. **c** Representation of fibers with varying diameters highlighting the atoms located at the surface layer (yellow). The surface layer is defined at the radial position where the fraction of Si3 significantly differs from that of the bulk. The surface layer has a fixed thickness, between 5 to 10 Å, regardless the fiber diameter. **d** Overall Si3 fraction as a function of the fiber diameter. At high diameters, the Si3 fraction tends to approach the bulk values, due to the low surface to volume ratio. At low diameters, the increasingly higher surface to volume ratio results in a pronounced increase of the fraction of Si3 (~ D.^−1^)

The simulated fibers apparently show a reasonably cylindrical cross-section, while for the thinnest fibers, some surface roughness is visible (see Fig. 2a). A reliable structural characterization of the fiber surface is essential for the understanding of the structure–mechanical property relations and the accurate estimation of the fiber cross section, specifically at high surface-to-volume ratios [12, 17, 27, 31, 39]. Here, to characterize the fiber’s atomic structure, the structural defects were computed along the fiber radial direction (refer to Supporting Information for additional details on density and structure analysis). This analysis was shown to provide a reliable information for the structural characterization of fibers and the surface [17, 27, 39].

Figure 2b shows radial distribution of the fraction of three-fold coordinated silicon atoms (Si3) as a function of the radial distance for both methods and varying diameters. The distribution reveals a low concentration of these species at the inner region, where the structure properties approach that of bulk glass. As moving towards the surface, the Si3 fraction increases considerably. The radial position where the Si3 fraction starts to increase defines the start of the surface region.

Noticeably, the surface layer is defined within a fixed thickness, between 5 and 10 Å, based on the silicon defect profile, regardless the fiber diameter (see Fig. 2c). Indeed, a similar surface thickness (6–7 Å) was reported by earlier MD simulations of silica fibers developed by Pedone et al. [39]. Therefore, the defect-rich surface layer becomes more and more dominant with decreasing fiber diameters, resulting in a noticeable increase of the overall fraction of Si3 (~ 1/*D*) with respect to bulk values (Fig. 2d). Other structural defects such as non-bridging oxygen (NBOs) were also analyzed and showed a similar profile (Fig S2).

To test the effect of interatomic potentials on the results, identical fiber simulations were performed using Teter [36] and Vashishta [37] potentials (see Section 2.1 for details of the potentials). The resulting Si3 and NBOs radial profiles reveal a similar surface thickness (Fig S3 and Fig S4), suggesting that the results are roughly potential independent. Also, as observed from the Si3 peaks, cut fibers surface layer is around 1 Å to 2 Å thicker as compared to that of cast fibers (Fig. 2b). Therefore, cut fibers are more defective than cast ones (Fig. 2c). Such findings are in agreement with earlier results of silica fiber simulations obtained by Yuan et al. [31]. In addition, the thickness of the defect layer on the surface is independent of the fiber diameter.

### Surface effects on tensile properties

Results of the previous section evidence the presence of a defect-rich surface layer whose effects become significant at the smallest diameters. Therefore, on the basis of current interpretations for the experimental BDT in glass nanofibers, which associate nanofiber ductility with surface effects [26–28, 43], an enhanced ductility would be expected at decreasing diameters, due to the higher atomic fraction of the defect-rich surface.

**Fig. 3:**
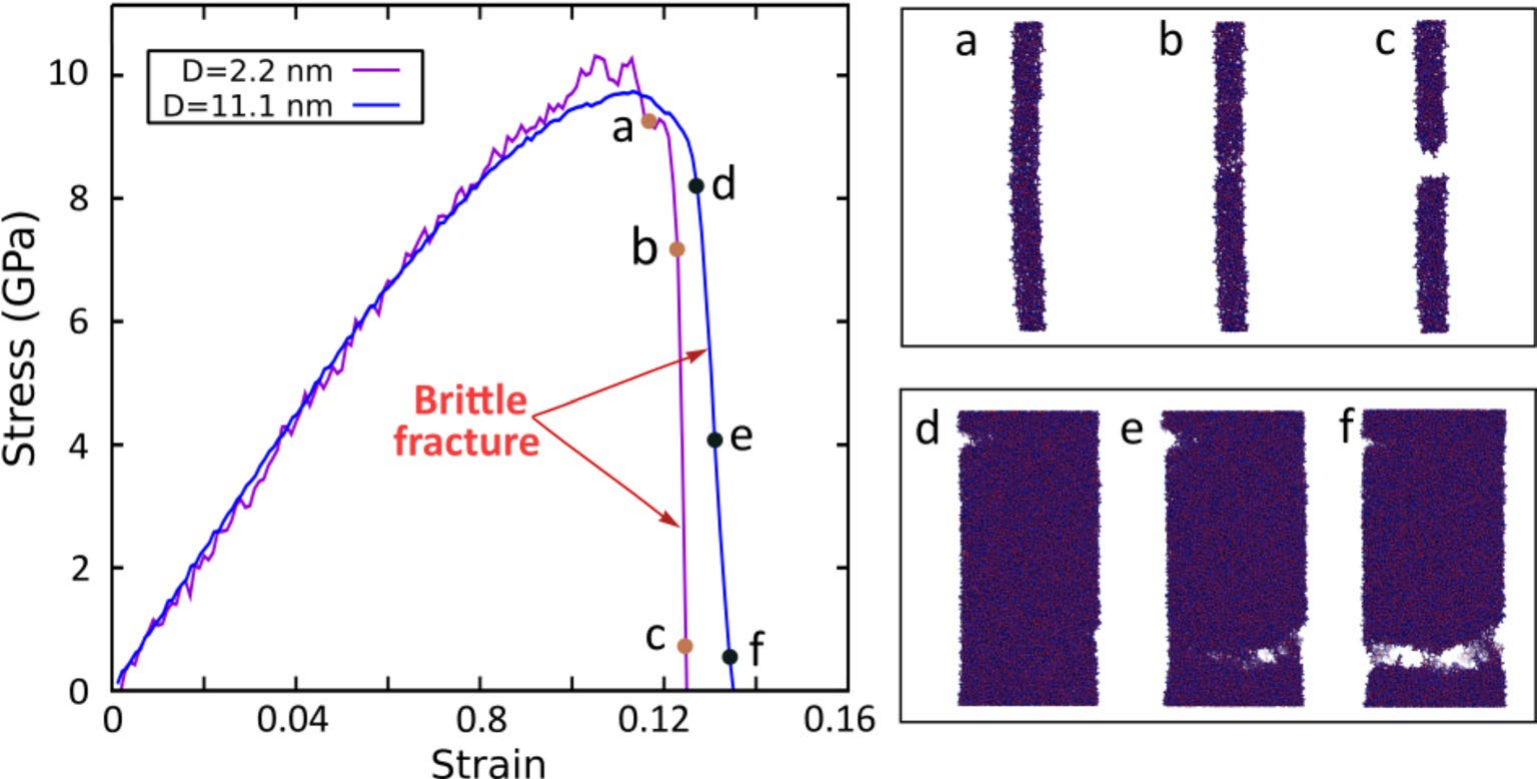
(Left) Stress–strain curves of cast fibers with a fixed length of 20.1 nm, and diameters of 2.2 nm and 11.1 nm obtained from DCRP potential together with (right) snapshots of different stages of the deformation process (see Movies S1 and S2 the full animations of the fiber deformation)

**Fig. 4:**
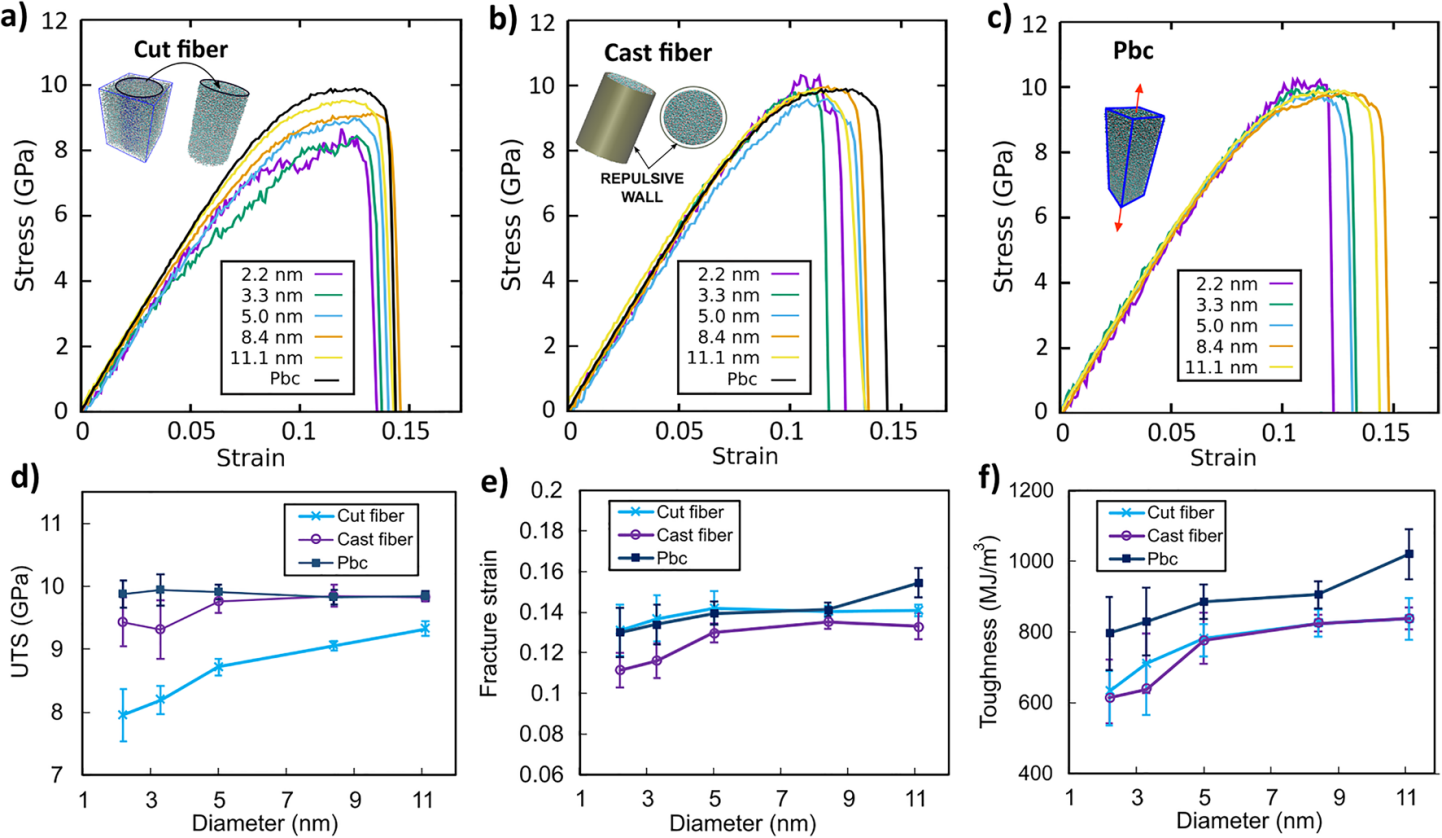
Stress–strain curves of representative fibers with varying diameters obtained using the **a** “cutting” and **b** “casting” production methods and **c** pbc structures (structures with periodic boundary conditions in all axes). Black curves represent the stress–strain curves of pbc samples with an equivalent diameter of 11.1 nm. Stress–strain curves characteristic quantities averaged over the results of all replicates as a function of the fiber diameter for the different production methods and pbc samples: **d** Ultimate Tensile Stress (UTS), **e** fracture strain and **f** toughness

To assess the effect of the surface layer on the fiber deformation, MD simulations of tensile tests were performed in the fibers and the resulting stress–strain curves were analyzed. Figure 3a compares the stress–strain curves of two fibers with the same length of 20.1 nm and reasonably different diameters. The sharp stress fall in the stress–strain curve suggests a brittle fracture and an absence of plasticity in both fibers. Indeed, both fail catastrophically by a sudden internal crack initiation followed by rapid crack propagation and eventual fracture (see Movies S1 and S2 for the fiber deformation animations). The abrupt stress fall is also observed for fibers formed using both methods at varying diameters (Fig. 4a, b).

Such findings of brittle fracture contrast with previous experimental results on silica glass nanofibers performed by Luo et al., who found a BDT as their diameter reduces below 18 nm [7]. Given the higher strain rates in MD simulations as compared to experiments, additional MD simulations of thin fibers at varying strain rates were performed, showing that brittle fracture remains at strain rates few orders of magnitude lower (see Fig S5 for results and discussion) To avoid the influence of this parameter, which is out of the scope of the present work, we performed all tests at a strain rate of 10^9^ s^−1^. In such conditions, the present results suggest that the increasing dominance of the surface at decreasing diameters (Fig. 2c) may not explain the BDT observed in glass nanofibers [7].

Further information on the role of fiber surface on the fracture behavior and mechanical properties can be obtained by generating structures with varying boundary conditions. To this end, equivalent structures with periodic boundary conditions in all axes (pbc structures) were tested (refer to Sect. 2.3 for computation details). Figure 4a–c shows the representative deformation curves obtained from a random structure of each sample size, as a function of the diameter, for cut and cast fibers, together with that of pbc structures (see Fig S6 for the complete set of curves). This initial analysis suggests that, in the yielding region, cast fibers and pbc samples show minimal variation in yielding strength across different diameters, whereas cut fibers exhibit a significant decrease in yielding strength as diameter decreases. Additional information about Young’s Modulus studies can be found in the Supporting Information.

To achieve more significance, results based on an average of different initial conditions are included (see Table 1). Figure 4d–f presents a comparison of the average values of the Ultimate Tensile Stress (UTS), failure strain and toughness (i.e. total area under the stress–strain curve) of both fiber models, together with bulk structures values, considering all the replicates of each sample deformation testing (see Table 1).

A lower UTS, combined with a higher fracture strain and toughness, is confirmed in cut fibers with respect to cast ones (Fig. 4d–f). These findings are consistent with the conclusions obtained by Yuan et al. [31]. Accordingly, they performed MD tensile simulations of silica fibers and found a decrease in the tensile strength and an increase of fracture strain in cut fibers compared to cast ones [31]. They attributed this effect to the highly defective surface layer of cut fibers, which reduce the network rigidity while impart flexibility to the nanofiber, therefore favoring larger deformations.

Meanwhile, with decreasing fiber diameters, a remarkable UTS decrease is observed in cut fibers compared to cast fibers, which remains barely constant (Fig. 4d). This effect may result from the increasing role of the highly defective surface layer of cut fibers, which inevitably leads to structure weakening [31, 39]. Moreover, with decreasing diameters, no increase of ductility is appreciated in the fibers as compared to their pbc counterparts (Fig. 4e). Indeed, they even show a toughness decrease (Fig. 4f), as opposed to the experimental results obtained by Luo et al. [7].

Overall, no significant improvements of fiber mechanical properties as compared to the bulk samples were observed. Particularly, despite cast fibers show a similar UTS to the bulk samples, they show a lower fracture strain. In contrast, cut fibers, despite showing a comparable fracture strain, have significantly smaller UTS. The interaction of these quantities results in fiber toughness values appreciably lower as compared to those of bulk samples. Similar conclusions have been obtained for simulations using the Teter and Vashishta potentials (Fig S7). These findings align with the observations of Bonfanti et al., who compared the deformation of cut fibers to their bulk counterparts with the aim to analyze the role of surface in the BDT. They reported a significant decrease in tensile strength in the cut fibers compared to the bulk samples, without a noticeable increase in strain [17].

Therefore, despite the increasing dominance of the surface layer at short diameters that results in defective samples (Fig. 2c), our MD simulations using two different fiber models do not support the idea that surface defects increases plastic flow thus ductile behavior during the fracture.

### Brittle-to-ductile transition

Altogether, our simulation findings challenge the prevailing interpretations of nanofiber mechanical properties, which typically attribute their increased ductility to surface effects [26–28, 43]. Specifically, Luo et al. suggested that the increasing role of the surface layer at decreasing diameters, where bond-switching events responsible for plasticity take place, may be the origin of the BDT [7]. Therefore, to elucidate the mechanisms involved in such transition, we further investigated the fiber length effect on the fracture behaviors [7]. Indeed, earlier MD simulations of bulk structures revealed a considerable effect of structure length on ductility [31, 32, 44, 45].

**Fig. 5:**
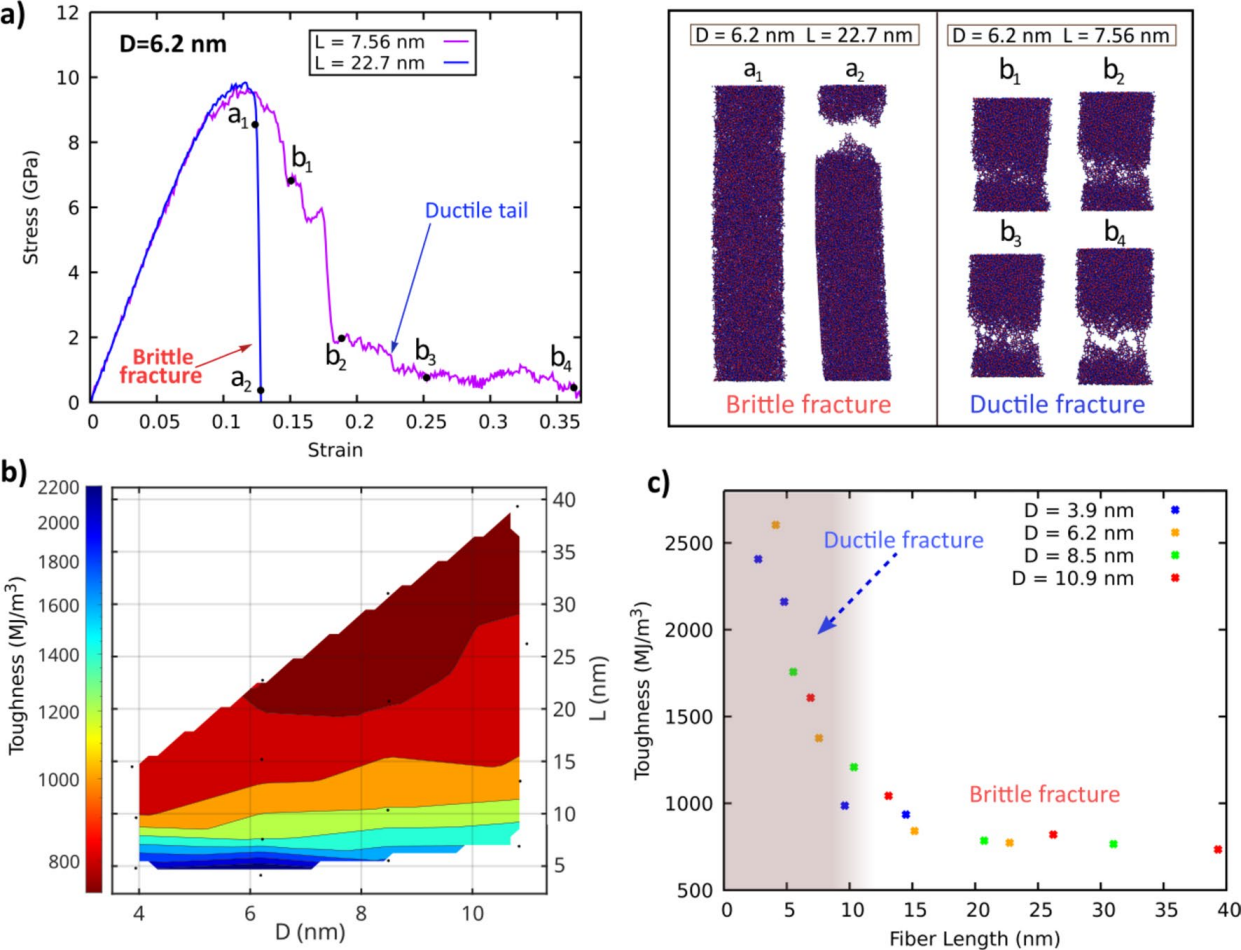
**a** Stress–strain curves of cast fibers with a fixed diameter of 6.2 nm and lengths of 7.56 nm and 22.7 nm together with snapshots of the fiber deformation states. The shortest fiber shows a large ductile tail combined with necking, while the largest fiber fails catastrophically (see Movies S3 and S4 for the full animations). **b** Diameter-length map of the toughness. The map reveals a major influence of fiber length in the ductile effects, which are favored by short length fibers. **c** Toughness as a function of the fiber length for varying diameters. The curve confirms the correlation with the fiber length, regardless their diameter. A BDT can be identified as the length decreases below 10 nm

To assess the length effects on plasticity, additional tensile simulations of fibers with varying diameters and lengths were performed. The length effect was considered in terms of the length-to-diameter ratio, *l*/*D*. Four different levels of the *l*/*D* ratio of 0.7, 1.25, 2.5, and 3.75 and diameters of 3.9 nm, 6.2 nm, 8.5 nm and 10.9 nm were considered (see Fig S8 for their stress–strain curves). Fig. 5a compares the stress–strain curves of two of them, with same diameter of 6.2 nm, and different lengths of 7.56 nm and 22.7 nm. Remarkably, while the largest fiber shows an abrupt failure, the shortest one exhibits a large ductile tail with signs of nanoductility, suggesting a major role of the fiber length on the BDT. A simultaneous study of the length and diameter effects reveals a minor role of fiber diameter on ductility (Fig. 5b). Instead a more pronounced effect of fiber length is clearly seen. Fig. 5c presents the resulting toughness values in terms of fiber length, for different diameters. The results show that ductility is favored by short length fibers, regardless their diameter, and a BDT is observed at lengths below 10 nm. These results also match with previous studies on MD simulations of tensile deformation of pure silica glass, which found that size effects only become relevant as the dimension is below 10 nm [31, 32, 45]. Above this value, the stress–strain curve turned roughly independent of structure dimensions [45].

The size-dependent fracture behavior and the role of surface effects were analyzed by Bonfanti et al., who compared different sample sizes of pbc samples and fibers [17]. Stress drop statistics of fiber deformation at 1 K revealed an increasing presence of "thermal-like" small stress drops at smaller diameters, which were associated with surface effects [17]. However, the fact that the pbc samples exhibit larger UTS while maintaining similar fracture strains compared to their equivalent fibers, and that small silica samples are ductile regardless of the presence of a surface, contributed to discount surface effects as a major factor leading to ductility [17].

Alternatively, it was shown that diffuse damage accumulation in small samples better explains the enhanced ductility [17]. Accordingly, the deformation occurs through localized bond breaks that affect the stress fields differently depending on the sample size [17, 46, 47]. When the glass dimensions are small, bond failure leads to large stress drops, and stress redistribution occurs due to the higher influence of the generated stress fields in smaller samples [17, 46, 47]. As a result, catastrophic failure can be prevented, and enhanced ductility can be achieved. In larger samples, stress drops are smaller, and the stress fields generated after bond failure do not significantly affect other regions. Therefore, structural accommodation does not occur, leading to brittle failure. Our simulation results are in excellent agreement with this theory: large ductile tails were observed for short length fibers while a sharp stress fall was observed for longer ones (see Fig S8).

### Analytical description of the BDT

Damage accumulation may provide reasonable explanation for the mechanisms involved in small size ductility [17]. However, this theory alone cannot provide a clear explanation for the onset of ductile processes. The reason is that long fibers can indeed accumulate a similar amount of damage as short fibers [17]. Alternatively, it is well known that fracture is favored whenever sufficient elastic energy is available for the creation of a fracture surface (fracture surface energy) [31, 32]. In this way, small samples store less elastic energy during deformation to promote fracture, leading to a ductile deformation. Meanwhile, larger samples store sufficient elastic energy that can easily stimulate fracture. Therefore, we propose an energetic interpretation describing the ductile processes in terms of two energy terms, the elastic strain and fracture surface energies (ESE and FSE) [31, 32]. This theory successfully explained the size effect of brittle-to-ductile fracture of bulk silica fibers [32].

**Fig. 6:**
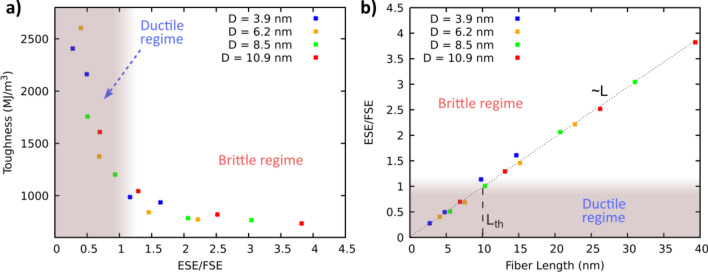
**a** Toughness versus elastic strain energy (ESE) to fracture strain energy (FSE) ratio. **b** ESE/FSE ratio versus fiber length (right) for different fiber diameters

We hence calculated both energy terms in the simulated fibers (refer to Supporting Information for energy calculations) and the results are summarized in Fig. 6. Figure 6a shows the toughness against the ESE/FSE ratio for varying fiber diameters. As observed, at high ESE/FSE ratios, low toughness and a brittle regime occurs. Conversely, as ESE/FSE decreases below a value of 1, an extraordinary increase of toughness is observed.

Therefore, it can be concluded that the ESE/FSE ratio can be an indicator of the BDT of the simulated fibers with the transition ratio being close to 1. It is worth mentioning that the ESE/FSE ratio increases linearly with fiber length (Fig. 6b), which allows the threshold length to be extracted, determining the onset of ductile processes.

Based on the analyses of fracture behavior of all simulated fibers, an analytical definition of the threshold length, *L*_th_, for the BDT onset, is proposed considering different properties of glasses. For this purpose, the ESE can be simplified as:

1 $$\text{ESE}=\frac{1}{2}E{\varepsilon }^{2}{A}_{\text{c}}l$$

where *E* is the Young’s Modulus, ε the elastic strain, A_c_ the fiber cross section *l* the fiber length. FSE is expressed as:

2 $$\text{FSE}=2{\upgamma }_{\text{f}}{A}_{\text{c}}$$

Being γ_f_ the unrelaxed fracture surface energy. Under such conditions, an ESE/FSE ratio of 1 results in *L*_th_ of:

3 $${L}_{\text{th}}=\frac{4{\gamma }_{\text{f}}}{E{\varepsilon }^{2}}$$

Below which ductile processes are expected to dominate [44]. By including the simulated values of the glass properties involved in the previous equation, a simulated value of *L*_th_ can be computed. γ_f_ is obtained from the fiber division and creation of two flat surfaces in absence of structural relaxation, equaling 2.78 J/m^2^ (refer to Supporting Information for fracture energy calculations), while *E* has a value of 109.6 GPa and ε of 10%, which approximately defines the limit of the elastic region. Based on these data, the predicted threshold length (*L*_th_) is 10.15 nm, in great agreement with Fig. 6b.

Unfortunately, using the values of γ_f_, *E*, and ε observed in MD simulations do not allow a direct correlation with experimental results, as variations of these values with respect to experiments are very common in MD simulations. However, an experimental threshold length can be estimated by using experimental values of these properties. Typical experimental value for γ_f_ is 3.7 J/m^2^ in a non-passivated and unrelaxed pure silica surface [48, 49]. Moreover, typical values for *E* and ε of commercial silica fibers are 70 GPa and 3%, respectively. Based on these experimental values, the predicted experimental threshold length yields a value of 230 nm, below which ductile fracture is expected to prevail in silica glass nanofibers.

Interestingly, the above predicted experimental threshold gauge length can successfully explain the BDT observed in the literature. For example, in an experimental study of dimension effects on fracture behaviors by Luo et al. [7], a SiO_2_ glass nanofiber with a diameter of 4.7 nm showed a ductile fracture with an elongation of ~ 18% while a 33.9 nm fiber shows a brittle nature with an elastic deformation of ~ 2.5%. This behavior can be explained by the fact that the gauge lengths of the thinner fiber (29 nm) is considerably lower than the estimated threshold length (of 230 nm), while that of the thicker fiber, of 350 nm, is higher. Thus, by leveraging these experimental values, the predicted threshold length provides a plausible explanation for the BDT observed in silica nanofibers, offering valuable insight into their fracture behavior.

## Conclusions

In conclusion, the deformation of amorphous silica nanofibers was studied through MD simulations to explore the role of fiber dimension and the surface layer on the deformation and fracture behaviors. The effect of structural defects in the surface layer was carefully analyzed by simulating nanofibers generated following two production models with different degrees of surface defects. The deformation curves were compared against that of bulk structures (i.e. absent of surface).

We show that surface defect effects do not enhance the fracture toughness of glass nanofibers. This was supported by the overall poorer mechanical properties obtained for the fibers with surface structural defects as compared to the bulk samples. On the contrary, the results show that an increasing role of the highly defective surface layer at decreasing diameters results in a UTS reduction. Therefore, toughness decrease with decreasing diameters was observed, revealing a negative influence of surface defects on fracture toughness contrary to some earlier theories that attributed ductility to surface defects induced plasticity.

Consequently, we propose that fiber gauge length may play a major role on fracture behaviors and better explains the BDT observed in glass nanofibers, both from simulations and experiments. We propose that the BDT can be defined by a balance between elastic strain energy stored during deformation and fracture surface energy. Such approach led to an analytical expression for a threshold length defining the BDT. Using experimental values of different properties of glass, such expression was used to estimate an experimental value for such length. Following this approach, our theory predicted a threshold gauge length of around 200 nm, below which ductile fracture dominates. This approach has been shown to successfully explain the BDT in glass nanofibers experimentally observed by Luo et al. [7].

Hence these results confirm the need to consider fiber testing gauge length as an important factor affecting the mechanical properties of glass nano- and microfibers, not only macroscopically, but also at the nanometric range. The results also suggest that glass nanofibers ductility is constrained to extremely small diameters and gauge lengths in the order of a few hundreds of nanometers. At larger scales, as the case for made-to-measure nano- and microfibers for macroscopic reinforcement applications, fracture corresponds to typical brittle behavior, showing a linear stress–strain curve until fracture. In case of brittle fracture, long fibers meet the conditions of the EVT probabilistic models when the volume of the representative element can be reduced to contain the stress field range, and temperature and strain rate of the tests are kept constant [1]. In this case, the strength depends on size and frequency of structural flaws and the onset of failure occurs when the critical flaw appears.

Finally, we acknowledge that the ductile behavior observed in short silica nanofibers, while interesting from a theoretical perspective, may not be ideal for reinforcing applications in nanocomposites. First reason is that the observed ductile tails in short silica nanofibers indicate a complete yield of the material, leading to a loss of their reinforcing effects. Secondly, to form fibers with such short lengths is not practical in real applications. Indeed, it is well known that maximizing surface defects and the aspect ratio (*l*/*D*) of nanofibers is crucial for effective load transfer from the matrix to the fibers, which is essential for enhancing the composite's mechanical properties. Nonetheless, this study aims to provide key theoretical insights into the mechanisms driving the mechanical behavior of these nanofibers, which can inform future material optimization.

## Supplementary Information

Additional file1 (DOCX 1426 KB)

Additional file2 (MP4 8075 KB)

Additional file3 (MP4 8092 KB)

Additional file4 (MP4 9804 KB)

Additional file5 (MP4 21198 KB)

## Data Availability

"Data sets generated during the current study are available from the corresponding author on reasonable request. Numerical data generated from the simulations are stored in private servers and can only be accessed by authorized users".

## Code availability

The data relevant to this article is not in a repository. Data included in this article is all presented.
