# Supplementary material for: Influence of fiber dimensions on the mechanical properties of silica glass nanofibers
Source: Discov Nano. 2025 Feb 14;20(1):37. doi: 10.1186/s11671-025-04210-0 (PMC11828773; doi:10.1186/s11671-025-04210-0)
Supplement: Supplementary file 1 — Additional file1 (DOCX 1426 KB) [file 11671_2025_4210_MOESM1_ESM.docx]

Supporting information

Influence of fiber dimensions on the mechanical properties of silica glass nanofibers

Raúl Barciela^1^, Félix Quintero^1,*^, Thiruvilla S. Mahadevan^2^, Antonio Riveiro^1,3^, Juan Pou^1^, Jincheng Du^2,*^

^1^CINTECX, Universidade de Vigo, LaserON research group, E.E.I, Vigo, 36310, Spain

^2^Department of Materials Science and Engineering, University of North Texas, Denton, Texas 76203, USA

^3^*Materials Engineering, Applied Mechanics and Construction Dpt., University of Vigo, E.E.I., Vigo 36310, Spain.*

^*^Corresponding authors: [Jincheng.Du@unt.edu](mailto:Jincheng.Du@unt.edu), [fquintero@uvigo.gal](mailto:fquintero@uvigo.gal).

**Precursor bulk structures preparation.** The precursor bulk silica structures were generated using a melt-quench process at a cooling rate of 5 K/ps following two steps. First step consists of an NVT cooling from 6000 K to 1300 K. Then, an NPT (constant number of atoms, pressure, and temperature) ensemble up to 300 K was performed. The NPT step was performed from an initial pressure of 100 bar, linearly decreasing to 1 bar at 300 K. Then, at 300 K, two subsequent equilibration steps of 100 ps at a constant pressure of 1 bar and 100 ps at constant energy were performed. For Vashishta and Teter potentials, the transition from NVT to NPT was performed at 3600 K.

**Density analysis.** The density study has shown that fibers obtained from cut method usually yield similar core density values as the corresponding to bulk structures. The core density of the cut fibers with the highest diameter has values of 2.332, 2.37 and 2.575 g/cm^3^, for DCRP, Teter and Vashishta, respectively, while that of bulk structures yields values of 2.331, 2.358 and 2.57 g/cm^3^, respectively, for each of the potentials. For the cast method, the density of the fibers with the highest diameter generated with DCRP and Teter potentials also yields similar values compared to that of bulk structures (2.316 and 2.357 g/cm^3^, respectively), while that of Vashishta potential, with a value of 2.644 g/cm^3^_,_ is reasonably higher.

**Structure analysis.** The structure analysis is performed by computing the atomic defects along the radial direction of the fiber. For this, different concentric cylindrical shells of a thickness of 0.5 Å were created, starting from the fiber z axis to a radial distance sufficiently far from the fiber surface. Then, the atomic defects are calculated in each shell. An analysis of the Q^n^ distribution of the generated fibers using the three potentials reveals that only Si3 and Si4 silicon atoms are present. Also, from the study of the oxygen species, apart from the typical presence of non-bridging oxygen atoms (NBOs), tetrahedrally-bridging oxygen atoms (TBOs) are also reported in all the fibers. Therefore, in the present simulations, the formation of Si3 implies the creation of NBOs and TBOs. The presence of oxygen peaks corresponding to TBOs has been difficult to assess experimentally in nuclear magnetic resonance [1, 2]. However, previous works on MD simulations of amorphous silica fibers also suggested the existence of TBOs [3] and it is proposed from MD simulations that they play an important role in topological hardening of more complex glasses as aluminosilicate glasses [2].

**Young’s modulus.** For pbc structures, Young’s modulus values of 109.9 GPa, 101.4 GPa and 125.4 GPa, were obtained for DCRP, Teter and Vashishta potentials, respectively. Noticeably, the Young’s modulus values are sensibly higher for cast fibers compared to cut ones, commonly to the three potentials. This effect results in Young’s modulus values which can be noticeably higher in case of cast fibers compared to pbc structures (for Teter and Vashishta) while more similar values are obtained in cut fibers (Teter and Vashishta). Overall, the Young’s modulus obtained in this work are sensibly higher compared to that obtained from experiments (72.9 GPa) [4], which can be related with the higher densities obtained with respect to the experimental value of 2.2 g/cm^3^ [4]. Indeed, these results align with the experimentally observed relationship between elastic moduli and glass density, where densified silica glass upon compression exhibit higher elastic moduli [5, 6]. The higher density and elastic moduli can be explained by the higher pressure (100 bar) involved during the quenching process of the precursor bulk silica structures. Lower densities and elastic moduli closer to experiments are expected from the simulated potentials when atmospheric (1 bar) pressure is used during the quenching process [7].

**Fracture surface energy calculations.** The total fracture surface energy of the fibers, $FSE_{\mathrm{unrelaxed}}$, was calculated by forcing the structure to break without any structural relaxation. For this, two flat surfaces were created by inserting a gap of 50 Å in *z* dimension in absence of structural relaxation. Then, fracture surface energy is computed as the difference between potential energy before inserting the gap and after inserting the gap [8–10].

$FSE_{\mathrm{unrelaxed}}$ represents the energy change in case of a perfectly brittle fracture takes place in the sample. Once the $FSE_{\mathrm{unrelaxed}}$ is estimated, the fracture surface energy, $\gamma_{f,unrelaxed}$, is calculated from the fiber cross section as $FSE_{\mathrm{unrelaxed}}$/*A*_c._

On the other hand, elastic strain energy, $ESE$, is computed based on the area covered by the elastic region of the stress-strain curve. The elastic region is commonly defined by the yield point, which corresponds to the point of the deformation curve leading to a non-recoverable strain of 0.2 % after stress release. However, in MD simulations of uniaxial tensile tests, a strong non-linear behavior is commonly observed, which turns this approach unrealistic for the definition of the elastic limit. As a result, elastic strain limit is taken as 10% [3, 11, 12].


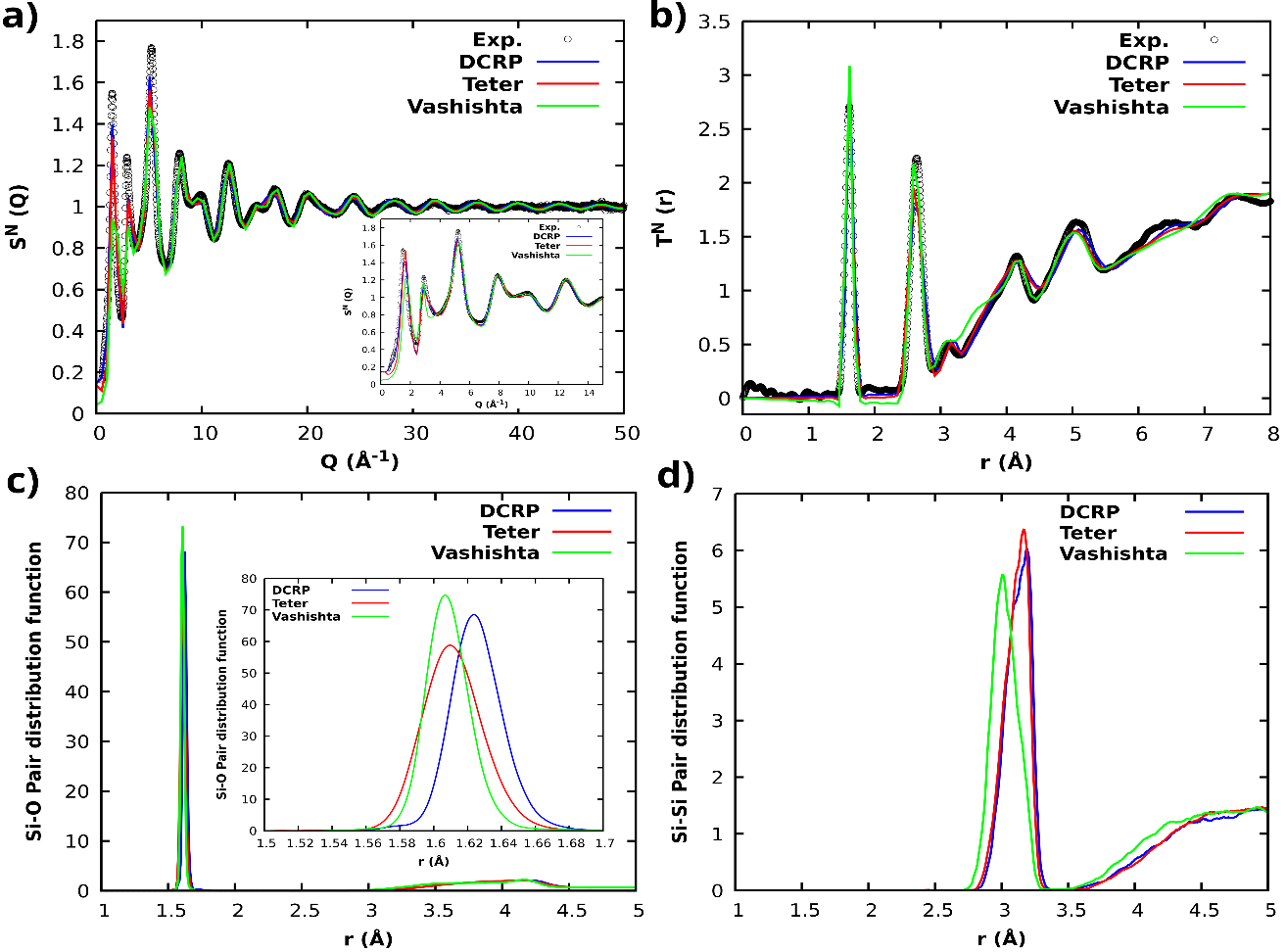


Fig S1. (a) Structure factors (S^N^ (Q)) and (b) total correlation functions (T^N^ (r)) resulting from the precursor bulk structures generated with the three potentials, compared to experimental results [13]. Pair distribution functions of (c) Si-O and (d) Si-Si bonds. As observed, in the higher reciprocal space, the structure factors do not exhibit a noticeable dependence on the potential and are consistent with neutron diffraction data. In the lower reciprocal space, the diffraction peak positions and intensities closely match the experiments for the DCRP and Teter potentials, while slightly lower intensities are observed for the Vashishta potential. Also, the total correlation functions reasonably match with the experiments. The Si-O pair distribution function yields first Si–O peak position values of 1.62 Å and 1.61 Å and 1.61 Å for DCRP, Teter and Vashishta, which are closed to the experimental value of 1.59 Å [14]. The first Si–Si peak positions are located within a distance of 3.19 Å and 3.17 Å and 3.01 Å for DCRP, Teter and Vashishta, respectively, also in good agreement with the experimental value of 3.061 Å [14].


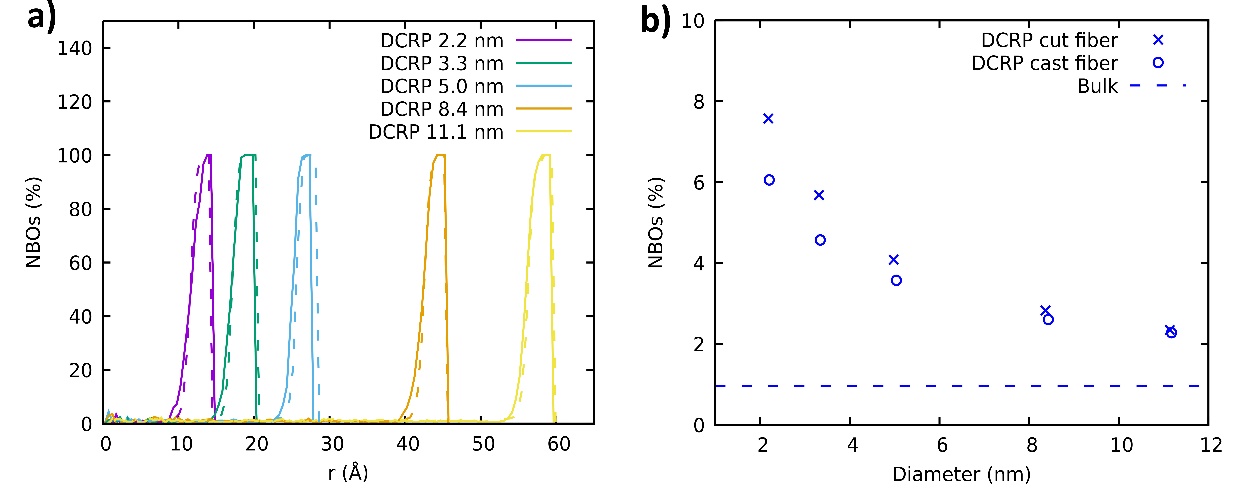


Fig S2. (a) Radial distribution of NBOs fraction for the “cutting” (dashed lines) and “casting” (solid lines) production methods and DCRP potential. (b) Overall NBOs fraction as a function of the fiber diameter. Dashed lines show the bulk value.


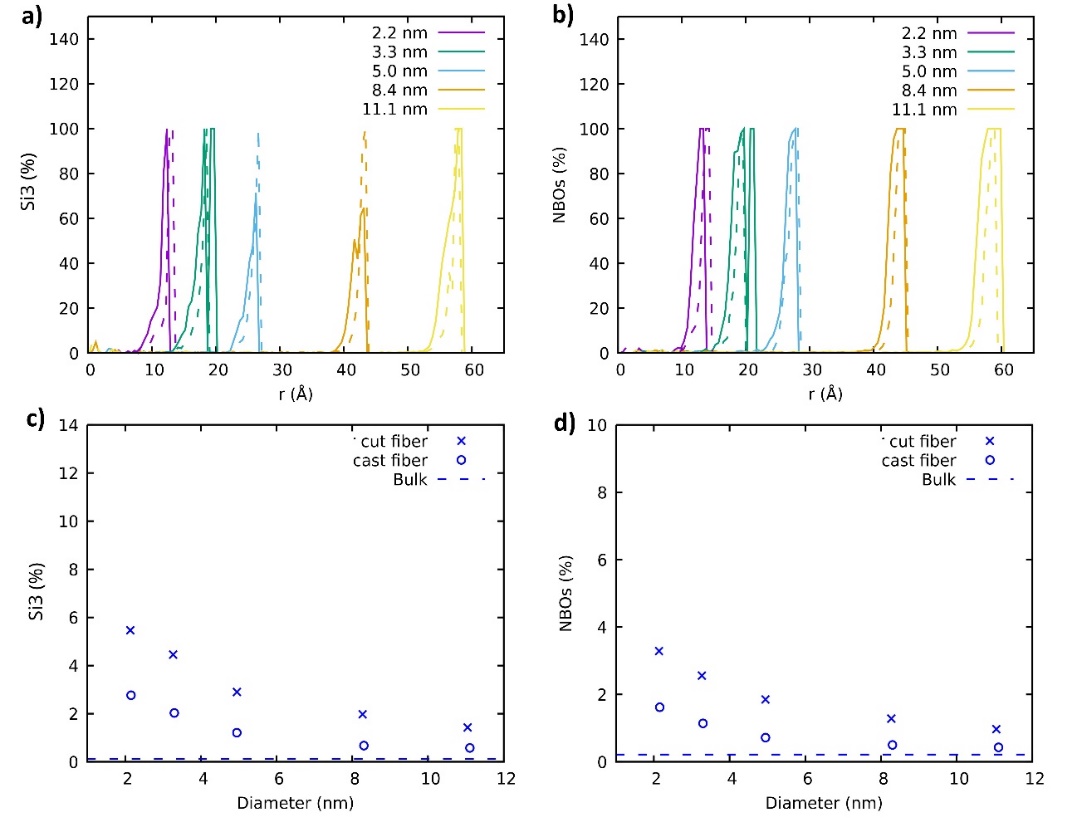


Fig S3. (a) Radial distribution of (a) Si3 and (b) NBOs fraction for the “cutting” (dashed lines) and “casting” (solid lines) production methods and Teter potential. Overall fraction of (c) Si3 and (d) NBOs as a function of the fiber diameter. Dashed lines show the bulk value.


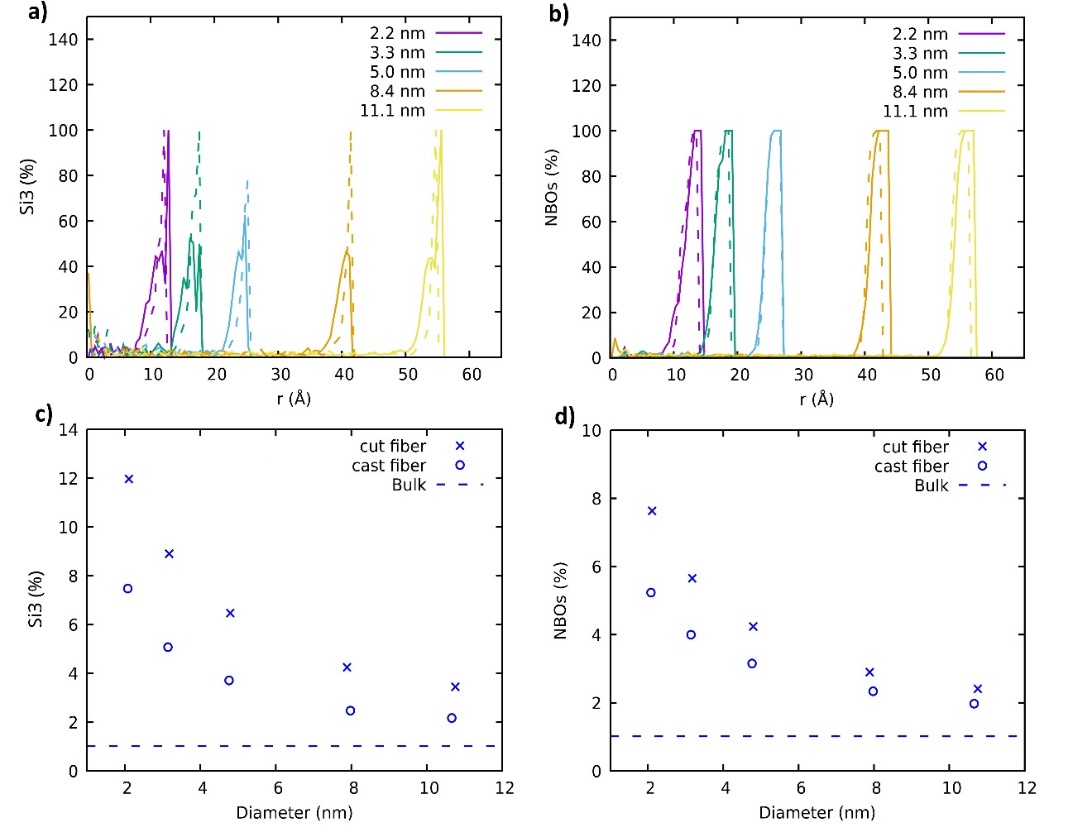


Fig S4. (a) Radial distribution of (a) Si3 and (b) NBOs fraction for the “cutting” (dashed lines) and “casting” (solid lines) production methods and Vashishta potential. Overall fraction of (c) Si3 and (d) NBOs as a function of the fiber diameter. Dashed lines show the bulk value.


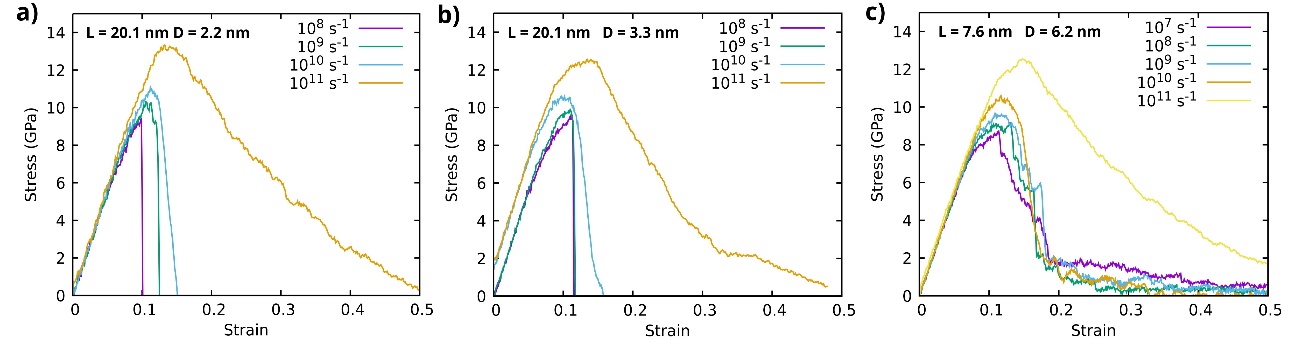


Fig S5. Stress-strain curves of cast fibers with varying dimensions L and D of (a) 20.1 nm and 2.2 nm, (b) 20.1 nm and 3.3 nm (c) 7.6 nm and 6.2 nm, at strain rates of different orders of magnitude. Simulations reveal decreasing ductility and strength by reducing strain rate in thin fibers with a length of 20 nm and diameters of 2.2 nm (a) and 3.3 nm (b). In fibers with a length of 7.6 nm, the ductile fracture is retained at low strain rates. The strain rate effects on ductility of glasses has been discussed in the literature [8, 9, 15–17]. MD simulations usually show a decrease of ductility at decreasing strain rates, which has been related with the longer time for voids to form and coalesce into critical voids[8, 9, 15]. In contrast, experimental works on tensile tests of silica nanofibers report the opposite behavior[16, 17]. The explanation of this behavior is that low strain rate may provide enough time for void recovering through bond-switching and relocation, leading to an enhanced plasticity. Such discrepancy between experiments and simulations, despite being a matter of debate, is out of the scope of this study. Moreover, the change in strength with strain rate raises the question of whether this could be the reason for the higher Young’s modulus observed. However, an analysis of the elastic portions of the stress-strain curves, which remain unaffected by strain rate variations, alongside similar findings from other studies [15], contribute to disregard strain rate as the factor responsible for the elevated Young’s Modulus attained. Instead, a stronger correlation with the quenching pressure is found (refer to Young’s Modulus discussion).


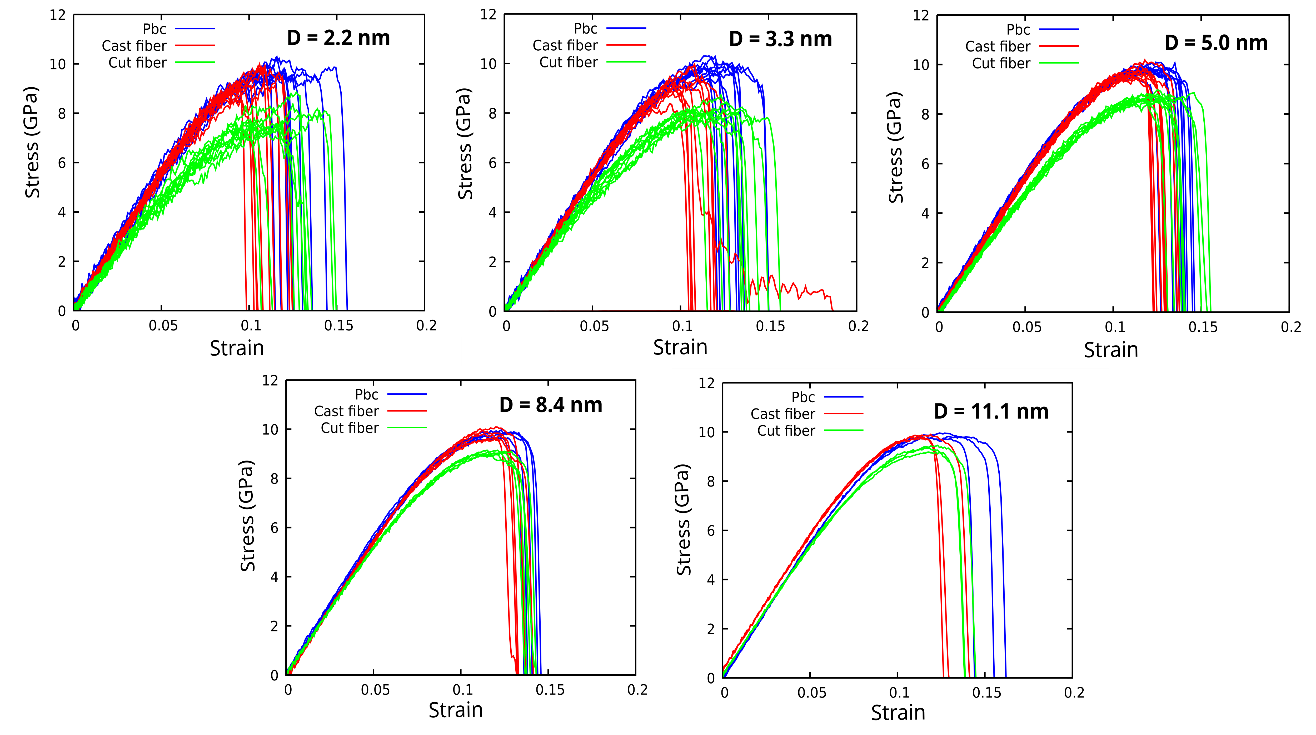


Fig S6. Stress-strain curves of samples with varying diameters obtained using the “cutting” and “casting” production methods and pbc structures. The sets of curves with identical color represent the results of all replicates of each size (see Table 1).


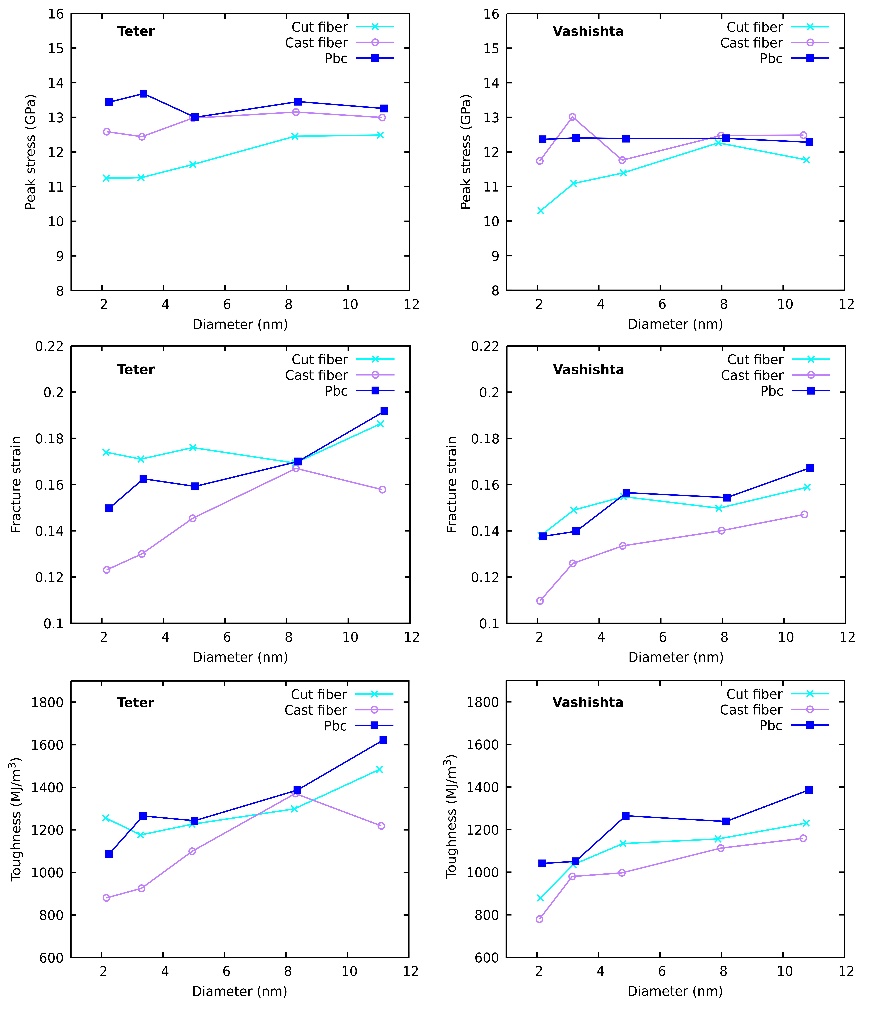


Fig S7. Stress-strain curves characteristic quantities as a function of the fiber diameter for the different production methods and Teter and Vashishta potentials: peak stress (upper panels), fracture strain (middle panels) and toughness (bottom panels). The corresponding values for pbc samples are also included.


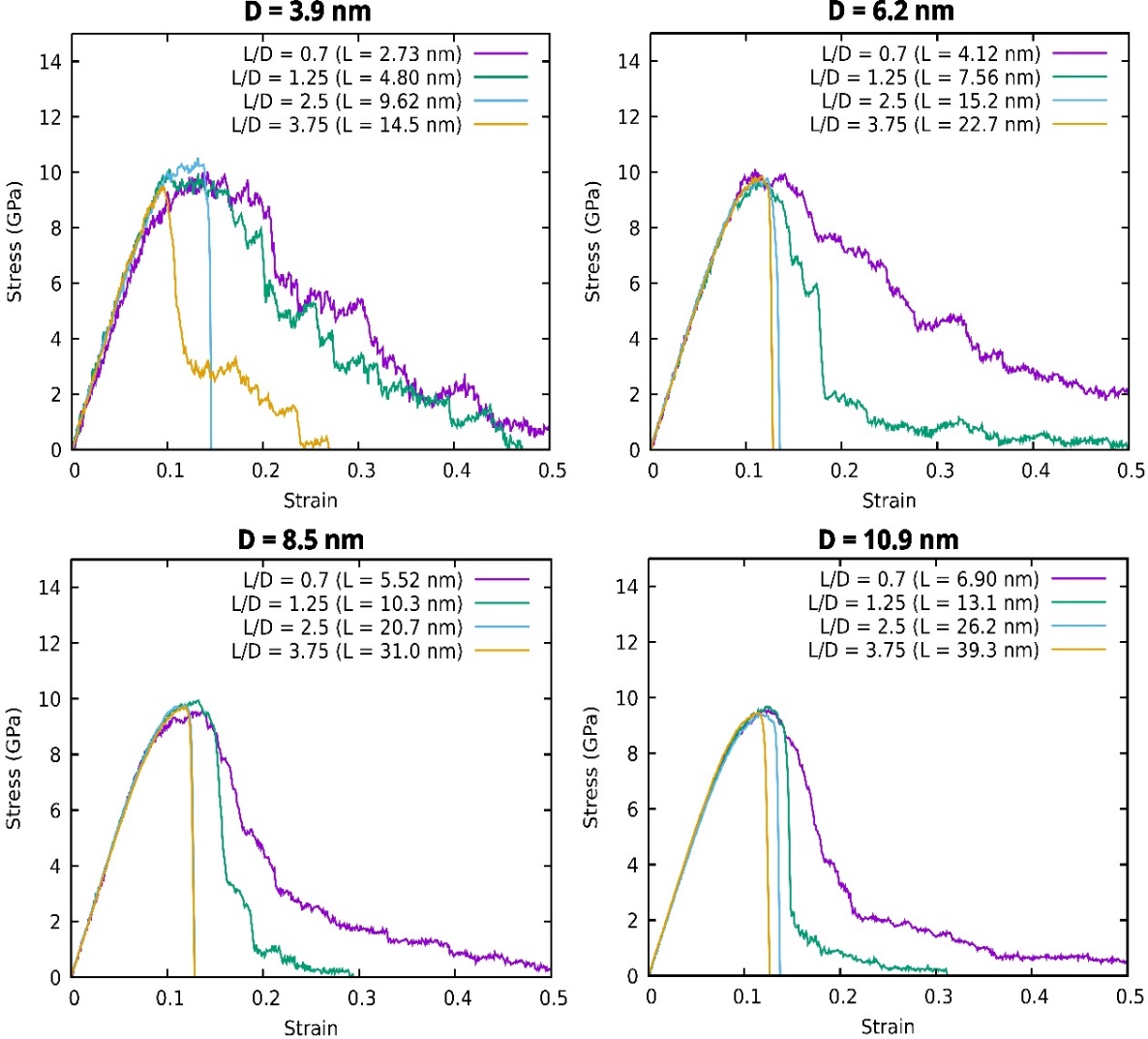


Fig S8. Stress-strain curves of fibers with fixed diameter and different lengths obtained following DCRP potential and cast method. The deformation curves in the elastic region until reaching the peak stress are notably similar for the different fiber sizes. Interestingly, fibers having the same diameter can exhibit a ductile tail provided that their length is sufficiently small. Conversely, when fiber length is large enough, a sharp fall in the stress-strain curve is observed after yielding, thus evidencing a change into brittle fracture behavior. Additionally, the deformation curve in the elastic region until reaching the peak stress does not differ between the different fiber sizes.

# Supplementary movies

- Supplementary Movie 1: Tensile deformation behavior of a thin silica nanofiber of a diameter D = 2.2 nm, shown in Fig 3 (a,b,c), evidencing a brittle fracture.
- Supplementary Movie 2: Tensile deformation behavior of a thick silica nanofiber of a diameter D = 11.1 nm, shown in Fig 3 (d,e,f), evidencing a brittle fracture.
- Supplementary Movie 3: Tensile deformation behavior of a large silica fiber, of a length L = 22.7 nm, and a diameter D = 6.2 nm, as shown in Fig 5, showing a brittle fracture.
- Supplementary Movie 4: Tensile deformation behavior of a short silica fiber, of a length L = 7.56 nm, and a diameter D = 6.2 nm, as shown in Fig 5, revealing clear signs of nanoductility.

# References

1. Stebbins JF, Xu Z (1997) NMR evidence for excess non-bridging oxygen in an aluminosilicate glass. Nature 390:60–62. https://doi.org/10.1038/36312

2. Welch RS, Lee KH, Wilkinson CJ, et al (2021) Topological hardening through oxygen triclusters in calcium aluminosilicate glasses. J Am Ceram Soc 104:6183–6193. https://doi.org/10.1111/jace.18032

3. Pedone A, Menziani MC, Cormack AN (2015) Dynamics of Fracture in Silica and Soda-Silicate Glasses: From Bulk Materials to Nanowires. J Phys Chem C 119:25499–25507. https://doi.org/10.1021/acs.jpcc.5b08657

4. Bansal NP, Doremus RH (2013) Handbook of Glass Properties. Handb Glas Prop 1–680. https://doi.org/10.1016/C2009-0-21785-5

5. Deschamps T, Kassir-Bodon A, Sonneville C, et al (2013) Permanent densification of compressed silica glass: A Raman-density calibration curve. J Phys Condens Matter 25:. https://doi.org/10.1088/0953-8984/25/2/025402

6. Deschamps T, Margueritat J, Martinet C, et al (2014) Elastic moduli of permanently densified silica glasses. Sci Rep 4:1–7. https://doi.org/10.1038/srep07193

7. Barciela R, Mahadevan TS, Quintero F, et al (2024) Size effects on the fracture behavior of amorphous silica from molecular dynamics simulations. J Non Cryst Solids 631:122935. https://doi.org/https://doi.org/10.1016/j.jnoncrysol.2024.122935

8. Yuan F, Huang L (2012) Molecular dynamics simulation of amorphous silica under uniaxial tension: From bulk to nanowire. J Non Cryst Solids 358:3481–3487. https://doi.org/10.1016/j.jnoncrysol.2012.05.045

9. Shi Y, Luo J, Yuan F, Huang L (2014) Intrinsic ductility of glassy solids. J Appl Phys 115:. https://doi.org/10.1063/1.4862959

10. Wang B, Yu Y, Lee YJ, Bauchy M (2015) Intrinsic nano-ductility of glasses: The critical role of composition. Front Mater 2:1–9. https://doi.org/10.3389/fmats.2015.00011

11. Cormack AN, Segre U, Menziani MC, et al (2008) Molecular Dynamics Studies of Stress−Strain Behavior of Silica Glass under a Tensile Load. Chem Mater 20:4356–4366. https://doi.org/10.1021/cm800413v

12. Zhang C, Duan F, Liu Q (2015) Size effects on the fracture behavior of amorphous silica nanowires. Comput Mater Sci 99:138–144. https://doi.org/10.1016/j.commatsci.2014.12.020

13. Grimley DI, Wright AC, Sinclair RN (1990) Neutron scattering from vitreous silica IV. Time-of-flight diffraction. J Non Cryst Solids 119:49–64. https://doi.org/https://doi.org/10.1016/0022-3093(90)90240-M

14. Trease NM, Clark TM, Grandinetti PJ, et al (2017) Bond length-bond angle correlation in densified silica - Results from 17O NMR spectroscopy. J Chem Phys 146:. https://doi.org/10.1063/1.4983041

15. Muralidharan K, Simmons JH, Deymier PA, Runge K (2005) Molecular dynamics studies of brittle fracture in vitreous silica: Review and recent progress. J Non Cryst Solids 351:1532–1542. https://doi.org/10.1016/j.jnoncrysol.2005.03.026

16. Yue Y, Zheng K (2014) Strong strain rate effect on the plasticity of amorphous silica nanowires. Appl Phys Lett 104:12–16. https://doi.org/10.1063/1.4882420

17. Luo J, Wang J, Bitzek E, et al (2016) Size-Dependent Brittle-to-Ductile Transition in Silica Glass Nanofibers. Nano Lett 16:105–113. https://doi.org/10.1021/acs.nanolett.5b03070
